# Supplementary material for: Artificial shelters and marine infectious disease: no detectable effect of the use of casitas to enhance juvenile Panulirus argus in shelter-poor habitats on a viral disease dynamics
Source: PeerJ. 2023 Mar 20;11:e15073. doi: 10.7717/peerj.15073 (PMC10035424; doi:10.7717/peerj.15073)
Supplement: Supplemental Information 2 — Number of total and diseased lobsters per site and sampling date on control sites and casita sites throughout experimental stage A, and on casita sites throughout experimental stage B. All diseased lobsters found during experimental stage B were culled. [file peerj-11-15073-s002.docx]

**Supplemental Table S1: Number of lobsters per site and sampling date.**

Number of total and diseased lobsters per site and sampling date on control sites and casita sites throughout experimental stage A, and on casita sites throughout experimental stage B. All diseased lobsters found during experimental stage B were culled.

|  | **Experimental stage A** | | | |  |  |  |  |  |  |
| --- | --- | --- | --- | --- | --- | --- | --- | --- | --- | --- |
|  | **Control sites** | | | | | | | | | |
|  | Site 1 | | Site 3 | | Site 6 | | Site 9 | | Site 10 | |
| Date | Total | Diseased | Total | Diseased | Total | Diseased | Total | Diseased | Total | Diseased |
| Nov 2009 | 33 | 0 | 4 | 0 | 7 | 0 | 11 | 1 | 6 | 3 |
| Mar 2010 | 23 | 4 | 1 | 0 | 10 | 0 | 19 | 2 | 1 | 0 |
| Jun 2010 | 12 | 4 | 0 | 0 | 13 | 2 | 3 | 0 | 0 | 0 |
| Sep 2010 | 15 | 6 | 7 | 1 | 16 | 2 | 8 | 1 | 3 | 1 |
| Dec 2010 | 10 | 1 | 10 | 1 | 6 | 0 | 2 | 1 | 3 | 0 |
| Feb 2011 | 6 | 1 | 4 | 0 | 7 | 1 | 5 | 0 | 5 | 1 |
| May 2011 | 7 | 2 | 3 | 0 | 9 | 1 | 8 | 1 | 2 | 0 |
| Sep 2011 | 14 | 6 | 4 | 0 | 15 | 0 | 6 | 0 | 2 | 0 |
| Jan 2012 | 15 | 3 | 10 | 1 | 23 | 1 | 5 | 0 | 1 | 0 |
| Apr 2012 | 14 | 4 | 5 | 1 | 22 | 1 | 20 | 4 | 0 | 0 |
|  | **Casita sites** | | | | | | | | | |
|  | Site 2 | | Site 4 | | Site 5 | | Site 7 | | Site 8 | |
| Date | Total | Diseased | Total | Diseased | Total | Diseased | Total | Diseased | Total | Diseased |
| Nov 2009 | 177 | 7 | 321 | 21 | 162 | 11 | 146 | 11 | 140 | 20 |
| Mar 2010 | 130 | 19 | 135 | 31 | 74 | 20 | 79 | 8 | 120 | 22 |
| Jun 2010 | 35 | 10 | 40 | 8 | 26 | 7 | 19 | 3 | 57 | 6 |
| Sep 2010 | 76 | 9 | 68 | 6 | 71 | 9 | 27 | 3 | 83 | 15 |
| Dec 2010 | 52 | 12 | 135 | 28 | 58 | 10 | 19 | 2 | 51 | 15 |
| Feb 2011 | 24 | 4 | 57 | 10 | 53 | 6 | 30 | 2 | 28 | 7 |
| May 2011 | 40 | 3 | 35 | 9 | 45 | 7 | 19 | 3 | 37 | 5 |
| Sep 2011 | 96 | 18 | 74 | 4 | 54 | 9 | 34 | 6 | 33 | 8 |
| Jan 2012 | 80 | 13 | 89 | 22 | 66 | 7 | 39 | 0 | 77 | 7 |
| Apr 2012 | 56 | 16 | 80 | 22 | 66 | 7 | 34 | 7 | 72 | 8 |
|  | **Experimental stage B** | | | |  |  |  |  |  |  |
|  | **Casita sites** | | | | | | | | | |
|  | Site 2 | | Site 4 | | Site 5 | | Site 7 | | Site 8 | |
|  | Total | Diseased | Total | Diseased | Total | Diseased | Total | Diseased | Total | Diseased |
| Date |  | (culled) |  | (culled) |  | (culled) |  | (culled) |  | (culled) |
| Sep 2012 | 32 | 7 | 98 | 22 | 39 | 12 | 32 | 9 | 44 | 11 |
| Jan 2013 | 151 | 28 | 83 | 25 | 36 | 4 | 40 | 4 | 48 | 9 |
| May 2013 | 50 | 12 | 70 | 18 | 32 | 4 | 13 | 1 | 25 | 8 |
| Sep 2013 | 79 | 17 | 111 | 26 | 107 | 20 | 43 | 1 | 74 | 14 |
| Jan 2014 | 41 | 11 | 21 | 6 | 88 | 14 | 55 | 10 | 70 | 17 |
| Jun 2014 | 38 | 9 | 36 | 8 | 62 | 11 | 35 | 3 | 40 | 10 |
| Sep 2014 | 34 | 10 | 36 | 13 | 53 | 8 | 49 | 11 | 70 | 9 |
| Jan 2015 | 31 | 11 | 26 | 9 | 33 | 3 | 25 | 8 | 45 | 9 |
